# Identification of Endogenous Reference Genes for RT-qPCR Expression Analysis in *Urochloa brizantha* Under Abiotic Stresses

**DOI:** 10.1038/s41598-017-09156-7

**Published:** 2017-08-17

**Authors:** Luciana Midori Takamori, Alyne Valéria Carrion Pereira, Gustavo Maia Souza, Luiz Gonzaga Esteves Vieira, Alessandra Ferreira Ribas

**Affiliations:** 1 Graduate Program in Agronomy, Plant Tissue Culture Laboratory, Universidade do Oeste Paulista (UNOESTE), Rod. Raposo Tavares, km 572, Limoeiro, 19067-175 Presidente Prudente-SP Brazil; 2 Plant Physiology Department, Federal University of Pelotas (UFPEL), Rua Almirante. Barroso, 1734 – Centro, 96010-280 Pelotas, RS Brazil

## Abstract

*Urochloa brizantha* is one of the most important warm season forage grasses in tropical countries. Despite its importance, there are few studies on gene expression in this species under stressful conditions. Real-time (RT-qPCR) is an accurate technique for gene quantification analysis, but reference genes must be validated under the same conditions used to assess the expression of the target genes. Here, we evaluated the stability of nine reference genes: Actin 12, Eukaryotic initiation factor 4 A, Elongation factor-1 alpha, FTSH protease 4, U2 auxiliary fator, Succinol Co-enzyme A, Tubulin alfa-5, Tubulin beta-6, Ubiquitin conjugating enzyme. Total RNA was extract from leaf tissues of *U. brizantha* subjected to 6, 12 and 24 h of cold and heat stresses (10 and 45 °C, respectively), and drought, including moderate (−0.5 to −0.7 MPa), severe (−1.1 to −1.8 MPa) and recovery after re-watering. The RefFinder web-based tool was used to rank the most stable reference genes for each stress. Elongation factor-1 alpha, Elongation factor-1 alpha or Ubiquitin conjugating enzyme, and Eukaryotic initiation factor 4 A were the most stable genes for heat, cold and drought stress, respectively. The expression of Rubisco large subunit gene was normalized against the most stable gene selected by ReFfinder for each stress.

## Introduction

Native from Africa, signal grass [(*Urochloa brizantha* (Hochst. Ex. A. Rich)] Stapf, formely *Brachiaria brizantha* ^1^ is a perennial grass belonging to the Poaceae family that is mainly used for livestock farming. Brazil is the second largest cattle and beef producer in the world. That production is based on planted pastures, as they provide feed and ensure low production costs^2^. Despite its great importance, this species is still considered a ‘genomic orphan’ due to limited availability of genetic and genomic information.

Quantitative real-time PCR (RT-qPCR) is considered the most accurate and reliable technique to measure gene expression and to validate data obtained by other methods like cDNA microarrays and RNA-seq. For some genes with very low levels of transcription, only RT-qPCR technique can detect such a small number of mRNA copies^3^. However, accurate normalization is a crucial step for the correct measurement of gene expression since the expression of constitutive genes might vary from cell to cell and according to the experimental conditions; therefore, the expression stability of candidate reference genes has to be verified before each research condition^4^.

Different algorithms have been developed to identify the best reference gene(s) among a group of potential candidate genes. NormFinder^4^, geNorm^5^, BestKeeper^6^ and ΔCt method^7^ are the most commonly methods used for this propose. These softwares calculate a measure of the stability of potential reference genes by comparing their individual stability in relation to the other tested genes under different experimental conditions. RefFinder^8^ is a web-based tool that integrates the four algorithms cited before. Based on the rankings from each program, it calculates the geometric mean to establish a comprehensive rank from a panel of candidate genes.

In *U. brizantha*, only one study investigated the expression profile of eight candidate reference genes in sexual and apomictic accessions of this species^9^. To our knowledge, there is no report regarding the identification of the stably expressed reference genes in *U. brizantha* for experimental conditions such as heat, cold and drought stresses.

In this work, we evaluate the suitability of nine reference genes [Actin 12 (*ACT12*), Eukaryotic initiation factor 4 A (*eIF4a*), Elongation factor 1-alpha (*EF1*-α), FTSH protease 4 (*FTHS*), U2 auxiliary fator (*U2AF*), Succinol Co-enzyme A (S*ucCoa*), Tubulin alfa-5 (α-*TUB5*), Tubulin beta-6 (*β-TUB6*) and Ubiquitin conjugating enzyme (*UbiCo*)] as candidates for normalization in RT-qPCR assays to study the transcriptional changes involved in the responses of *U. brizantha* to three different abiotic stresses using the RefFinder web-based tool^8^.

In addition, considering that Rubisco activity is highly regulated in response to abiotic stresses^10^, the relative expression of the plastidial large subunit of ribulose 1, 5-bisphosphate carboxylase/oxygenase (*rbCl*) gene in leaves of *U. brizantha* plants under the abiotic stresses was calculated to assess the reliability of the chosen reference genes to normalize the RT-qPCR data.

## Results

The description of the nine genes tested in this work and their genebank accession number are in Table 1. Primer sequences, amplicon sizes and amplification efficiency values are in Table 2. The primer efficiency was calculated with LinRegPCR software, where 2.0 means 100% efficiency (Table 1).

**Table 1:** List and description of the genes used in this study. Primer sequences (5′–3′) employed in the RT–qPCR analysis, amplicon sizes and LinRegPCR-derived PCR efficiency values.

Gene Name	NCBI genbank Accession number	Gene symbol	Forward primer	Reverse primer	Amplicon size (bp)	Primer efficiency ± SD	R
Actin 12	JG436709.1	*ACT12*	GGGTGGAGAGAGATTGCAGGTTA	GGGAACTGAGGCAACCACAGA	85	1.99 ± 0,073	0,9861
Eukaryotic initiation factor 4 A	EZ000622	*eIF4a*	CAGGTGTCCCTGGTCATCAACTAT	GAAACGACCTCTACGACCAATGC	81	2.01 ± 0,025	0,9998
Elongation factor-1 alpha	EZ000623	*EF1-α*	CAAGGCTGCTGCCAAGAAGA	CTGCAAGAACCAGCCTTGAACA	111	1.97 ± 0,052	0,9887
FTSH protease 4	JG437233.1	*FTSH*	GCAACTTATGGACCAGGAGGTT	CGCACTGGGTTCATCAATCA	115	1.97 ± 0,007	0,9995
U2 auxiliary fator	JG436537.1	*U2AF*	CGCCCACCTGCAAAGATATT	GGTGCTGGTAAGCAGTCCTCAT	104	1.90 ± 0,015	0,9984
Succinol Co-enzyme A	GE617476	*SucCoa*	ATGTACTTTGCCATCACCCTTGA	TCAATACTGGTTCCTCCCTTGCT	80	1.94 ± 0,029	0,9992
Tubulin alfa-5	GE617477	*α-TUB5*	CGTGTGCATGATCAGCAACA	GCGCTTGGCGTACATCAGAT	85	1.98 ± 0,019	0,9765
Tubulin beta-6	JG436975.1	*β-TUB6*	GTACCAGGACGCAACTGCTGAT	GAAATCTGCCTGCCCTAGAAGCT	119	2.07 ± 0,018	0,9989
Ubiquitin conjugating enzyme	GE617481.1	*UbiCo*	CATCTGCTCCCTGCTGACTGA	GCCGGTCCGTCTTGTACATATG	80	1.92 ± 0,049	0,9247
Rubisco large subunit from *U. Panicoides*	HE573318.1	*rbCl*	CGGAGTACGAAACCAAGGATACTG	ACTGCAGCCCCTGCTTCTT	91	1.97 ± 0,007	0,9994

**Table 2:** Ranking of candidate reference genes according to stability values in a pool of *Urochloa brizantha* leaves samples subjected to various abiotic stresses.

Gene Name	geNorm		NormFinder			BestKeeper		ΔCt				RefFinder	
	Rank order	Average expression stability (M)	Rank order		Stability Value (SV)	Rank order	Standard deviation [ + /−CP]	Rank order	Average of standard deviations		Rank order		Geometric mean of ranking values
**Heat Stress**													
*EF1-α*	6	1,297	1	0,727		5	1,21	1	1,35	1			2,34
*U2AF*	2	0,813	4	0,988		2	0,78	4	1,44	2			2,38
*β-TUB6*	1	0,813	8	1,472		1	0,76	8	1,73	3			2,83
*Act12*	5	1,231	2	0,788		6	1,46	2	1,39	4			3,31
α-*TUB5*	3	0,976	5	1,009		3	1,12	5	1,47	5			3,87
*eIF4a*	7	1,374	3	0,943		8	2,02	3	1,41	6			4,74
*SucCoa*	4	1,109	6	1,018		4	1,19	6	1,49	7			4,90
*FTSH*	8	1,453	7	1,360		9	2,35	7	1,68	8			7,71
*UbiCo*	9	1,529	9	1,517		7	1,77	9	1,80	9			8,45
**Cold Stress**													
*EF1-α*	1	0,667	4	0,592		1	0,84	3	1,18	1			1,86
*UbiCo*	3	0,824	1	0,501		4	0,91	1	1,15	2			1,86
α-*TUB5*	2	0,667	2	0,549		5	0,90	2	1,16	3			2,11
*eIF4a*	4	0,915	3	0,591		6	0,98	4	1,18	4			4,12
*SucCoa*	5	1,010	5	0,992		8	1,24	5	1,40	5			5,62
*Act12*	7	1,109	8	1,360		2	0,87	8	1,55	6			5,66
*FTSH*	8	1,206	7	1,247		3	0,90	7	1,55	7			5,66
*β-TUB6*	6	1,105	6	1,237		9	1,52	6	1,54	8			6,64
*U2AF*	9	1,392	9	1,496		7	1,00	9	1,73	9			8,45
**Drought Stress**													
*eIF4a*	1	0,565	1	0,656		6	0,96	1	1,26	1			1,57
*U2AF*	2	0,565	2	0,761		3	0,92	2	1,31	2			1,86
α-*TUB5*	5	1,208	5	1,155		1	0,74	6	1,54	3			3,50
*SucCoa*	6	1,261	3	0,772		4	0,93	3	1,33	4			3,83
*β-TUB6*	4	1,110	6	1,210		2	0,78	5	1,53	5			3,94
*EF1-α*	3	1,023	8	1,301		5	0,95	7	1,61	6			5,38
*Act12*	7	1,324	4	0,867		7	1,03	4	1,36	7			5,29
*FTSH*	8	1,414	7	1,294		8	1,11	8	1,64	8			7,74
*UbiCo*	9	1,474	9	1,437		9	1,51	9	1,68	9			9,00

**Figure 1:**
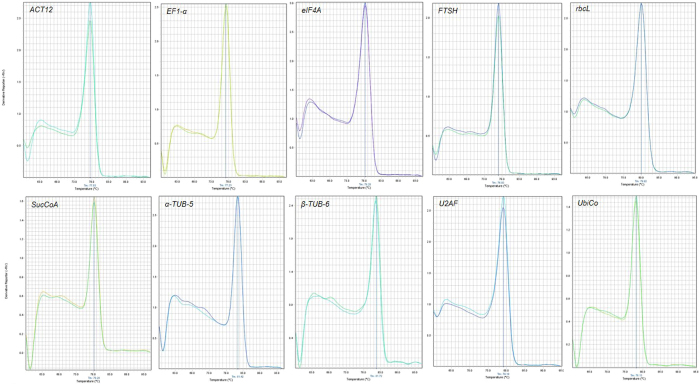
Melting curves of PCR products from all reference genes used in this study showing a single peak (each includes two technical replicates of the cDNA pool from all samples).

**Figure 2:**
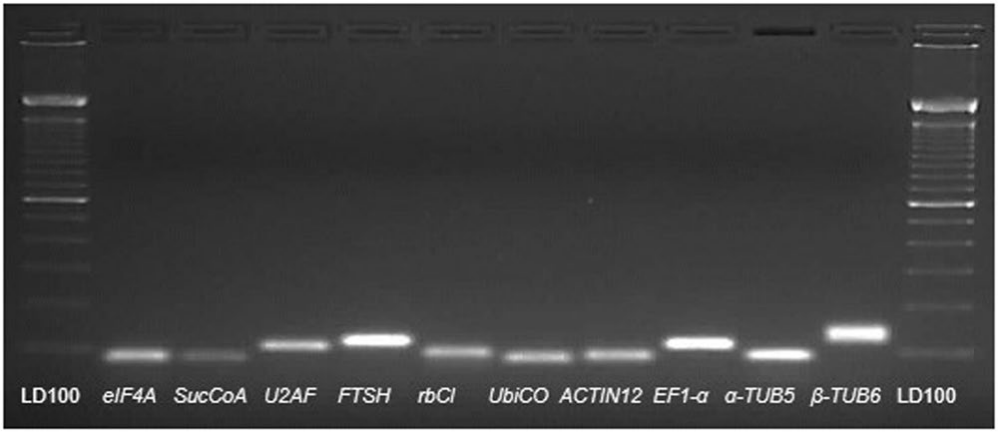
Ethidium bromide-stained agarose gel (1.2%) loaded with PCR products from cDNAs for each gene showing single amplificafion band.

The mean efficiency values for the primer pairs (three replicates) ranged from 1.90 (*U2AF*) to 2.07 (*β-TUB6)* that means 95 to 103.5% efficiency, respectively. The melting curve analysis performed at the end of RT-qPCR reactions for all selected candidate genes to check the specificity and integrity of the PCR products showed the presence of a single peak (Fig. 1). In addition, regular PCR reaction using the primer pairs followed by agarose gel electrophoresis revealed a single DNA band in each gel lane (Fig. 2).

**Figure 3:**
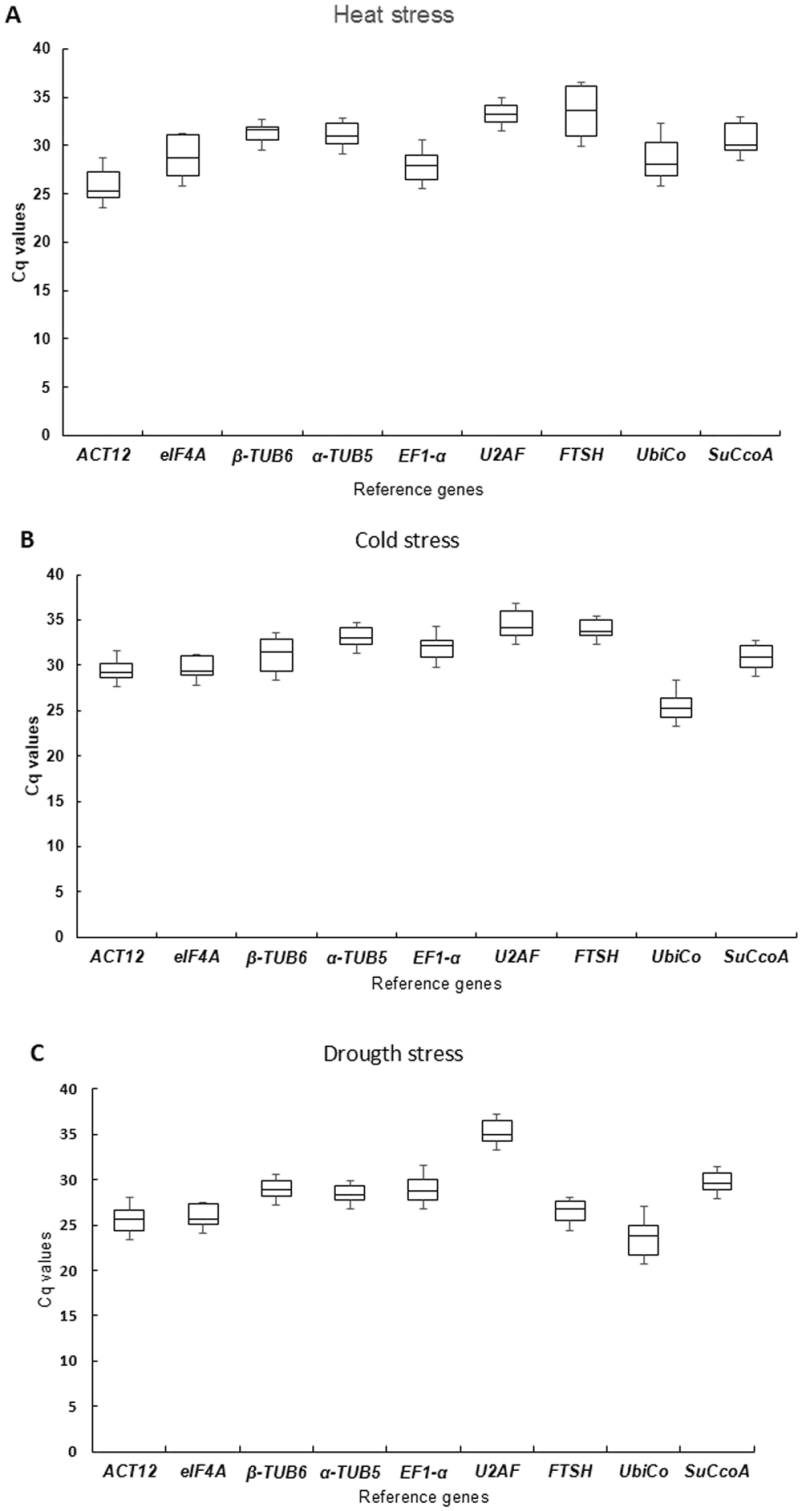
Average of Cq values of the candidate reference genes for each stresses: A –heat, B- cold and C – drought stress, with a total of 12 samples for each gene. Box-plot graph shows the median values as lines across the box. Lower and upper boxes indicate the 25th percentile and 75th percentile, respectively. Whiskers represent the maximum and minimum values.

**Figure 4:**
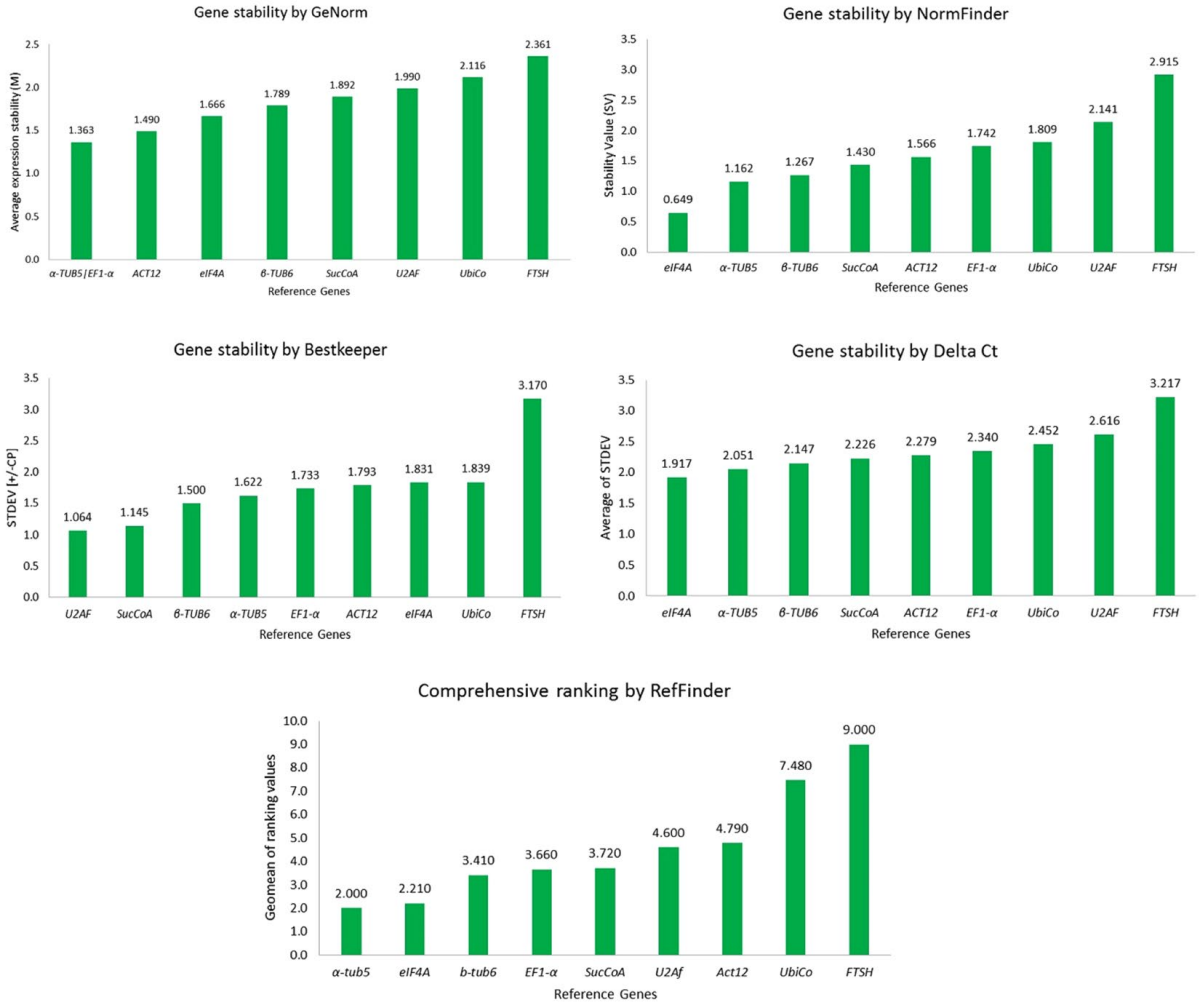
Rank of the candidate genes for all treatments combined (heat, cold and drought) generated by RefFinder web-tool showing the values for each of the four different algorithms. ΔCt method (Mean SD), NormFinder (Stability value), geNorm (Average expression stability M), BestKeeper (SD [±CP] crossing point values) and the final comprehensive ranking.

In order to select the best stable genes for normalization, expressions of the selected candidate reference genes were compared using leaf tissue of *U. brizantha* cv. Marandú plants under three abiotic stresses: drought, cold and heat shocks. The means and ranges of the Cq values are an indication of the most stable reference genes across all samples for each stress. The Cq means for all genes ranged from 22.8 (*Ubico*) to 34.4 (*U2AF*) for drought, 24.9 (*UbiCo*) to 33.7 (*U2AF*) for cold and 24.3 (*ACT12*) to 33.0 (*FTHS*) for heat stress. The lowest Cq values were 19.6 (*UbiCo*) for drought, 23.7 (*UbiCo*) for cold and 22.5 (*ACT12*) for heat stress, while the highest values were 36.5 and 35.9 (*U2AF*) for drought and cold stress, respectively, and 35.9 (*FTSH*) for heat stress (Fig. 3). The coefficients of variation (CV%) of the Cq values ranged from 2.89 (*U2AF*) to 8.2% (*UbiCo*) for heat stress, 3.4 (*EF1α*) to 5.5% (*β-TUB6*) for cold stress and 2.9 (α-*TUB5*) to 7.8% (*UbiCo*) for drought stress. To calculate the gene stability values of reference genes for gene expression studies in *U. brizantha* we used RefFinder^8^, a freely available web-based tool that combines the results from geNorm, NormFinder, and BestKeeper and ΔCt methods. When all stresses were considered together the NormFinder and ΔCt methods showed exactly the same classification for all genes from the most to least stable gene: (*eIF4a* > *α-TUB5 > β-TUB6 > SucCoa > ACT12 > EF1-α > UbiCo > U2AF > FTSH)* (Fig. 4). On the other hand, for the BestKeeper and geNorm algorithms only the two least stable genes ranked at the same position (Fig. 4). Considering all stress together α-*TUB5* was the most stable gene, while the reference gene *FTSH* was considered the least stable gene in all four algorithms and in the comprehensive ranking generated by RefFinder (Fig. 4).

The rankings of the four algorithms (geNorm, NormFinder, and BestKeeper and ΔCt method) and the RefFinder comprehensive ranking for each stress separately is shown in Table 2. The most and the least stable genes do not rank at the same position for all four algorithms. For heat stress, the most stable gene ranked by the NormFinder and ΔCt methods was *EF1-α*, while *β-TUB6* was selected by the geNorm and BestKeeper. *UbiCo* was ranked as the least stable reference gene, with exception of the BestKeeper software that selected *FTSH* instead.

For cold stress *EF1-α* was ranked as the most stable by geNorm and BestKeeper while *UbiCo* by NormFinder and ΔCt. For drought stress, *eIF4a* was ranked as most stable for all methods, except for BestKeeper; under this stress condition, the *UbiCo* was ranked as the least stable by the four algorithms (Table 2). Considering the comprehensive rank generated by RefFinder for each stress separately, the most stable genes were *EF1*-α, *EF1*-α or *UbiCo* and *eIF4a* for heat, cold and drought stresses, respectively, whereas the genes ranked as the least stable were *UbiCo* (heat and drought) and *U2AF* (cold) (Table 2).

**Figure 5:**
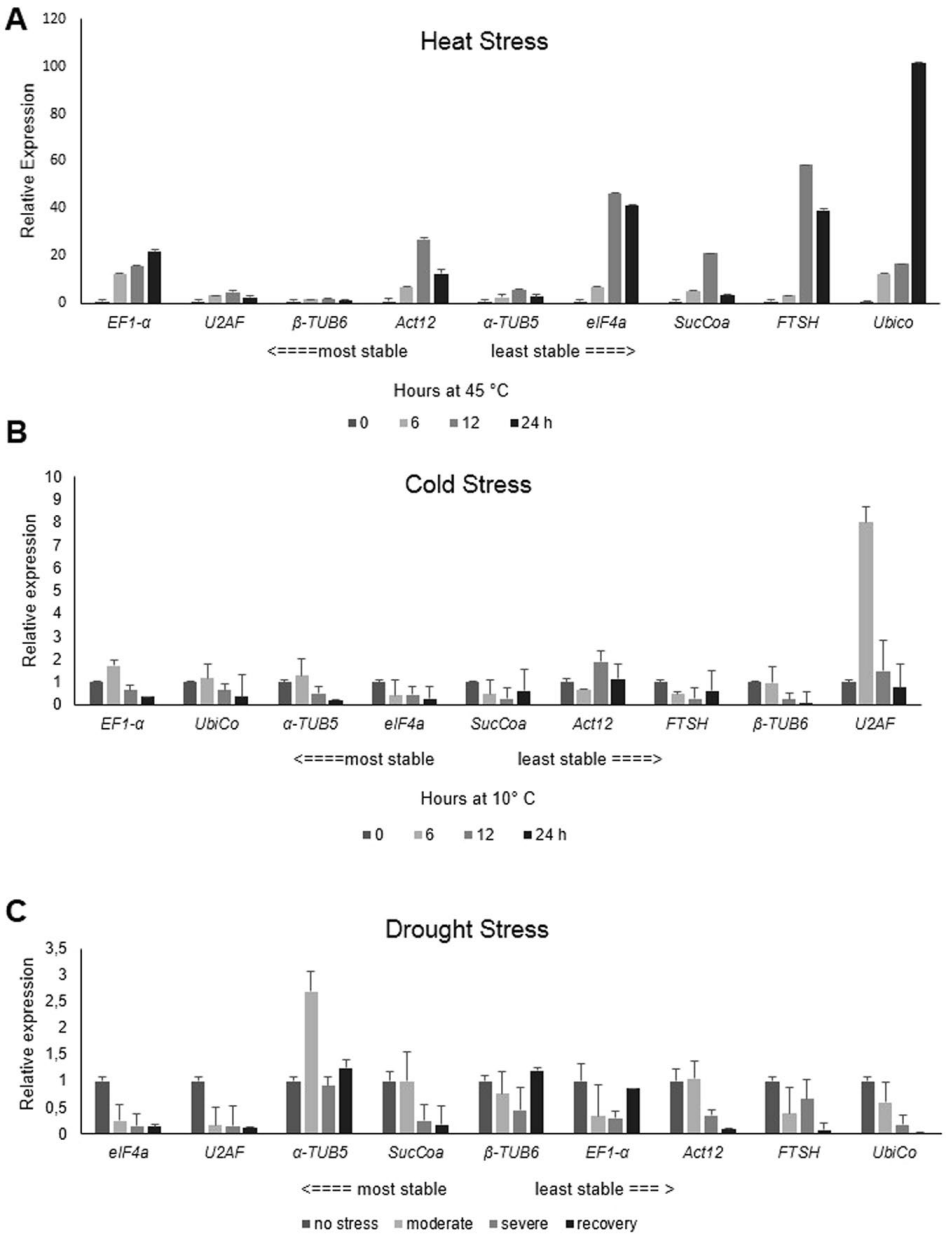
Relative expression of the *rbCl* gene in leaves of *U. brizantha* cv. Marandú under three abiotic stresses normalized to reference genes ranked according to the RefFinder approach: A – heat, B – cold and C - drought. The results are expressed as mean fold in change in relative expression compared to control samples. Bars indicate the standard error (±SE) calculated from three biological replicates.

The analysis of the transcriptional profile of the *rbCl* gene in leaves of *U. brizantha* cv. Marandú subjected to the various abiotic stresses was normalized against all genes from the most to the least stable gene selected by the ranking generated by RefFinder (Table 2). The results in Fig. 5 indicate that different reference genes can influence target gene relative expression levels. Our results showed that *EF1*-α was progressively upregulated under heat stress. However, when using the least stable gene (*UbiCo*) for that same stress treatment, the mRNA level showed a 5-fold difference in the severe heat stress condition (24 h at 45 °C) compared to the value detected using *EF1*-α (Fig. 5A).

For the cold stress treatment, the relative expression patterns of *rbCl* gene were very similar when normalized with the three most stable genes ranking by RefFinder (*EF1*-α, *UbiCo* and α-*TUB5*). In contrast, when normalized against the least stable gene (*U2AF*), the target gene *rbCl* showed a quite different expression pattern. When the *EF1*-α gene was used as reference, a 1.5 fold increase in transcript abundance of the *rbCl* gene after 6 h at 10 °C was detected, while a near 8-fold difference was observed when the transcripts were normalized against *U2AF* (Fig. 5B).

On the other hand, there was no noticeable difference between the *rbCl* expression in plants exposed to drought stress treatments when the data was normalized against the two most (*eIF4a* and *U2AF*), or the least stable gene (*UbiCo*), for the severe water decifit and recovery conditions. Although all algorithms, including RefFinder, ranked *UbiCo* as the least stable reference gene for normalization in leaves of *U. brizantha* submitted to drought stress (Table 2), the *rbCl* gene was shown to be slightly down-regulated across all water deficit treatments, similarly to the results obtained using the most stable reference genes (Fig. 5C).

## Discussion

RefFinder^8^, an easy to use web-based tool that integrates the currently most used methods (geNorm, NormFinder, BestKeeper and the comparative ΔCt method) was used to compare and rank a set of nine reference genes for assessing gene expression in *U. brizantha* plants under three abiotic stresses: heat, cold and drought. The geNorm determines the average pairwise variation (V) for a candidate reference gene in comparison with all other tested reference genes by calculating the expression stability measure (M). The NormFinder software compares each gene to the mean derived from the dataset to identify the gene(s) with the greatest stability^5^. BestKeeper directly uses Cq values as the input to calculate the geometric and arithmetic mean, minimal and maximal value, standard deviation and coefficient of variance^6^. ΔCt method approach compares relative expression of pairs of genes within each sample^7^. Comparisons among these methods have been frequently reported and, in many cases, a set of candidate genes selected by those approaches does not rank at same position^11–15^.

Based on the rankings obtained from each method, RefFinder gives an appropriate weight to individual genes and calculates the geometric mean of their weights for the overall final ranking^8^. The composite scores from RefFinder allow for the validation of the most stable reference genes. In this work, only the NormFinder algorithm and the ΔCt method gave the same rank order when considering all stresses together.

From the set of reference genes tested, tubulin alfa-5 (*α-TUB5*) was found to be the most stable while *FTSH* ranked as the least stable gene across all stresses (drought, cold and heat) *in U. brizantha* as calculated by RefFinder. This gene (*α-TUB5*) is one of the most commonly used as reference gene for the analysis of relative transcript expression levels; neverthless it was considered one of the most unstable reference among 32 genes used for quantification of gene expression in wheat in various tested conditions, including different tissues, developmental stages and temperature stresses^16^. Tubulin was also found to be the second most stable for normalization of different tissues of swichtgrass plants (*Panicum virgatum*) considering different abiotic stresses (drought, high salinity, cold, heat, and waterlogging) altogether. However, when each stress was analyzed separately, this gene was not ranked in the same position, presenting the largest variation in transcript levels between the stresses^17^.

As the expression of most the reference genes varied under different abiotic stresses, no single gene could be considered suitable for normalization of all experiments. Thus, it is more appropriate to select the reference genes for each specific stress. In this work, the eukaryotic initiation factor (*eIF4a*), which mediates the binding of mRNA to the ribosome, functioning as a subunit of the initiation factor complex *eIF4a* ^18^, was considered the most stable gene under drought stress using RefFinder. The *eIF4a* has also been identified as the most suitable reference gene for normalization in gene expression studies of sugarcane under drought treatments^13^. For different abiotic stresses, including drought, salt and heat waterlogging and ABA treatments, the *eIF4a* gene ranked as one of the best reference genes for normalization of RT-qPCR data in perennial ryegrass (*Lolium perenne*), although not always ranked first for all stresses^19^.

Eukaryotic elongation factor 1 alpha (*EF1-α*), which catalyzes the binding of aminoacyl-trna to the A-site of the ribosome by a GTP-dependent mechanism^20^, was identified as the most stable reference gene for both heat and cold stresses by the RefFinder tool. In several species and at different experimental conditions, *EF1-α* has been selected as one of the most stable genes. For example, it was considered the most stable gene in *Caragana intermedia*, a drought-resistant desert shrub, subjected to cold and salt stress^12^. In addition, for gene expression normalization in coffee plants under abiotic stresses, *EF1-α* was one of the recommended reference genes^21^.

In several species of grasses, the *EF1-α* gene has been selected as one of the most stable genes under different conditions. In perennial ryegrass, *EF-1α* and *UBQ5* genes were the most stably expressed reference genes in leaves during plant development^22^. This same combination of reference genes were found to be a reliable for RT-qPCR data normalization in aerial tissues of *Lolium temulentum* plants under abiotic stresses^23^. In male and female reproductive tissues, spikelets, roots and leaves of *U. brizantha*, the genes *EF1-α* and *UbiCo* were the more stable, while for ovary tissues, where apomictic and sexual reproduction occur, the best ranked reference genes were *UbiCo*, *eIF4a* and *EF1-α* ^9^.

The genes *EF1-α* and *eIF4a* are among the most stable reference genes for normalization in different monocot plant species, such as maize under heat and cold stress^24^ and switchgrass^25, 26^. In *Brachypodium distachyon*, the *EF1-α* was the most stable gene for cold and heat treatments^27^. Also, in leaves of creeping bentgrass (*Agrotis stolonifera*) under cold stress, *EF1*-α and *Ubi3* (E3 ubiquitin protein ligase) were selected by RefFinder as the most stable genes for normalization^28^. Besides that, *EF1-α* and RNA POL II were identified as the most appropriate internal controls for dehydration stress-related expression analyses in foxtail millet (*Setaria italica*)^29^.

In our work, as observed in other grass species, the *EF1*-α gene was ranked by RefFinder at the first position in gene stability for cold and drought stresses in leaves of *U. brizantha*. Ubiquitin-conjugating enzyme, ranked in the second position for cold stress, was also one the most suitable reference genes across different cold treatments in switchgrass^17^. Elongation factor-1 alpha and Ubiquitin-conjugating enzyme E2 were also ranked as the most stable genes across individual and multiple abiotic stresses, and also various developmental tissues in pearl millet (*Pennisetum glaucum*)^30^. On the other hand, *EF1-α* was considered the least stable gene for four abiotic stresses (drought, salt, dark and heat) in *Reaumuria soongorica*, a plant that is tolerant to extremely drought conditions, while the histone *H2A* and eukaryotic initiation factor 4A-2 (*eIF4a*) were considered the most stable genes for those conditions^31^.

Aiming to validate the reference genes selected for studies with *U. brizantha* plants under the three major abiotic stresses (heat, cold and drought) we evaluated the relative expression of the plastidial *rbCl* gene normalized to reference genes according to RefFinder rank. The plastidic *rbCl* gene encodes the large subunit of the primary CO_2_ fixation enzyme Rubisco in the mature C_4_ leaves of *U. brizantha* and has been used to validate candidate reference genes in plants submitted to abiotic stress^31^.

When normalization of *rbCL* gene was performed with reference genes poorly ranked according to RefFinder, a clear over-estimation of transcript abundance the target gene was observed in some samples, particularly in leaves subjected to heat and cold stresses. For drought stress, a high variation on expression pattern was observed depending on the reference gene used for normalization, except for the two better-ranked genes that showed exactly the same profile.

The analysis of the transcriptional profile of the *rbCl* gene in plants of *U. brizantha* cv. Marandú normalized with *EF1*-α, ranked as the most stable gene, showed that it was progressively upregulated under heat stress. C4 plants, such as *U. brizantha*, are generally adapted to warm environments. This fact could be explained by the increased CO_2_ assimilation rates at high temperatures at the place of Rubisco in the photosynthetic bundle sheath^32^. The increased transcript levels of large subunit of Rubisco was also observed during heat stress in tobacco, a C3 species, where increased respiration did not cause a significant change in photosynthesis^33^.

The analysis of the transcriptional profile of the *rbCl* gene in plants of *U. brizantha* cv. Marandú normalized with *EF1*-α showed that this gene was progressively upregulated under heat stress. This observartion was expected since C4 plants, such as *U. brizantha*, are generally adapted to warm environments. This fact could be explained by the increased CO_2_ assimilation rates at high temperatures at the place of Rubisco in the photosynthetic bundle sheath^32^. The increased transcript levels of the large subunit of Rubisco was also detected during heat stress in tobacco, a C3 species^33^.

For the cold treatment, the relative expression of *rbCl* normalized against the *EF1-α* showed only a slight increase in the transcript abundance after 6 hour at 10 °C and then a small but progressive decline until 24 °C of cold stress. This small variation in *rbCl* transcrips was expected since only in long-term acclimation the large subunits of rubisco increased in abundance^34^. It occurs because plants grown at low temperatures have higher amounts of photosynthetic enzymes, including Rubisco to compensate for decreased activities of the enzymes at low temperatures^34^. In contrast, when the *rcbl* gene was normalized against the less stable reference gene (*U2AF*), a large variability was observed, illustrating the low expression stability when using this gene for normalization of data from cold stress treatment.

The relative expression of *rbCl* gene against the two most stable reference genes *eIF4a* and *U2AF* showed the same profile. The *rbCl* gene was down-regulated under drought stress treatment. There was no remarkable expression differences between the most and the least stable candidate references used for normalize *rbCl* gene in *U. brizantha* subjected to drought stress. The *rbCl* was down-regulated when *U. brizantha* plants were exposed to the drought stress treatments using either the most stable or the unstable reference gene. In agreement with our results, the quantity of *rbCl* mRNA transcript was reduced when *Pinus halepensis* plants were subjected to drought stress^35^. This was expected as Rubisco was proved to be down-regulated in several stressful conditions that impose alterations in photosynthesis rate and carbon assimilation^32, 36, 37^.

The use of the stable reference genes resulted in the consistency of *rbCl* gene expression according to the literature, as discussed above. In contrast, biases were produced when the less stable reference genes were used as internal control, which could lead to misinterpretation of the *rbCl* expression patterns in *U. brizantha* plants under different environmental stresses.

To our knowledge, this is the first detailed report on selection and validation of reference genes for RT-qPCR of *Urochloa brizantha* plants under different abiotic stresses. Our results showed the stability of nine selected genes by using RefFinder to rank the potential candidate reference genes. Based on this approach, α-*TUB5* was ranked as the overall most suitable reference gene across the multiple abiotic stresses tested - drought, cold and heat. However, different reference genes are recommended for gene expression normalization for *U. brizantha* under those stress conditions: *EF1-α* for heat, *EF1-α or UbiCo* for cold and *eIF4a* for drought stress. In contrast, the frequently used reference genes *UbiCo* (for drought and heat) and *U2AF* (for cold) were the least suitable for the conditions used in this work. The appropriateness of these genes for further studies of abiotic stresses in *U. brizantha* was confirmed by the expression the *rbCl* gene after normalization.

## Methods

### Plant Material

Signal grass seeds from the 2012/13 season kept in cold storage at 15 °C were sown in trays containing Bioplant® substrate. Forty days after sowing, the seedlings were transferred to pots containing 5 kg of sandy soil (Ferric alisol). Soil acidity was corrected with dolomitic limestone and treated with 10-10-10 (NPK) fertilizer. The plants were cultivated in a greenhouse under natural light and long-day photoperiod of 16/8 h (light/dark) at 28 °C. The plants were irrigated daily with 500 ml of tap water.

### Cold and heat stresses

The stress treatments started five weeks after transferring the plants to pots. Initially, the pots containing two plants each were transferred to a phytotron (Eletrolab model EL011, São Paulo, Brazil), where they were kept for one week at 25 °C/day and 22 °C/night with a photoperiod of 16-h light (intensity 400 µmol photons m^−2^ s^−1^) and 8-h dark. The pots were irrigated every day to the maximum soil retention capacity.

The temperatures treatments were carried out after the plants were held for one week under the conditions described above. Sixteen pots containing two plants each were used for each experiment. Four pots were used as control (normal phytotron temperatures prior to stress treatments). After collecting the samples from the control plants, the other twelve pots were subjected to constant temperature of 10 or 45 °C for 24 h for cold or heat shock, respectively. For all treatments, each sample were constituted by six pooled young flag leaves collected before the beginning of each stress and after 6, 12 and 24 h under stressful conditions.

### Drought stress

This experiment was carried out under the same conditions depicted above for the initial growth of the plants in a greenhouse. Twenty pots, with two five weeks-old plants per pot, were used in this experiment. The plants were kept at 100% field capacity for a week and then exposed to four watering regimes: 1) No stress (soil water-holding capacity); 2) moderate stress (soil water content maintained at 50% of the maximum soil retention capacity until reaching −0.5 to −0.7 MPa); 3) severe stress (soil water content maintained at 20% of the maximum soil retention capacity −1.0 to −1.8 MPa); and 3) recovery (full-irrigation to soil saturation following the severe stress). The soil moisture was measured daily using sensors (model EC-TM Decagon devices, Pullman, USA). Leaf water status was monitored with a pressure chamber instrument (model PMS 1000, Oregon, USA). The light intensity at the green house was about 900 µmol photons m^−2^ s^−1^ during the day.

Biological replicates were represented by three pots with two plants each. Each sample was composed by a pool of six flag leaves collected from each pot subjected to the different stress levels. The samples were placed in plastic bags, frozen with liquid N_2_ and stored at −80 °C until RNA extraction.

### RNA extraction and cDNA synthesis

Each leaf sample (30 mg) was ground in liquid N_2_ using a mortar and pestle to a fine powder and quickly transferred to pre-cooled eppendorf tubes for RNA extraction. The total RNA was isolated using RNA Mini Kit Purelink™ (Invitrogen, Carlsbad, CA) and the samples were treated with TURBO DNAse™ (Life Technologies, Carlsbad, CA) according to the manufacturer’s instructions. After DNAse treatment, the samples were purified with phenol/chloroform, precipitated with isopropanol and resuspended in 20 µl of DEPC (diethylpyrocarbonate) water. The quality and quantity of total RNA were assessed with a Nanodrop ND-1000^TM^ spectrophotometer (Thermo Fisher Scientific, USA). Only RNA with OD 260:280 ratios always greater than 1.8 was used. The integrity of the RNA was checked upon electrophoresis in a 1.2% agarose gel with Tris-Borate-EDTA buffer (pH 8,0) stained with ethidium bromide by evaluating the integrity of the 28 S and 18 S ribosomal RNA bands and absence of smears.

For each sample, two micrograms of total RNA were used to synthesize first-strand cDNAs using oligo-(dt)18 primers and the Superscript III™ RT kit (Invitrogen, Carlsbad, CA), according to manufacturer’s recommendations.

### Selection of reference genes and primer design

Genbank accession numbers for *U. brizantha* ESTs (Expressed Sequence Tags) corresponding to the reference genes have been previously published by Silveira *et al*.^9^. These genes are Elongation factor-1 alpha (*EF1-α*), Eukaryotic initiation factor (*eIF*4a), Splicing factor (*U2AF*), FTSH protease 4 (*FTSH*), Succinyl-coa ligase (*SucCoa*), Tubulin alfa-5 (*α-TUB5*) and Ubiquitin conjugating enzyme (*UbiCo*). The accession numbers were used to search for nucleotide sequences against dbest database in the GenBank, which in turn, were used to design the primers. To search for ESTss of Actin 12 (*ACT12*) and Tubulin beta-6 (*β-TUB6*) genes of *U. brizantha*, sequences of switchgrass (*Panicum virgatum* L.) were used as query (accession numbers GR878265 and GR880018, respectively)^11^. The protein sequences of these genes were blasted against the dbest of *U. brizantha* using TBLASTN tool^37^. The selected ESTs were then used as query for the BLASTX (http://blast.ncbi.nlm.nih.gov/Blast.cgi) against the non-redundant protein database for identity verification. The gene description and the GenBank accession numbers of the ESTs used in this work are in Table 1.

For reference gene validation we design primers using the sequence from the plastidial large subunit of Rubisco (*rbCl*) homolog from *U. panicoides* (Accession number HE573318.1) (Table 1) because of the absence of *rbCl* nucleotide sequences from *U. brizantha* in the NCBI and the high conservation of this gene across plant species^38^.

All primers were designed using Primer Express software version 3.0.1 (PE Applied Biosystems, Foster City, USA) with the following parameters: amplicon length between 80 and 120 bp; melting temperature (Tm) between 58 and 60 °C, GC content ranging from 50% to 60% and length between 18 and 24 bases (Table 1). The specificity of the primers was checked using Primer-Blast tool (available at: (http://www.ncbi.nlm.nih.gov/tools/primer-blast/).

### RT-qPCR

The RT-qPCR assays were performed in 96-well plates using the StepOnePlus^TM^ Real Time PCR System (Applied Biosystems, Foster City USA). All cDNAs were diluted 1:25 to serve as a template for the reactions. Each RT-qPCR reaction contained 5 µl Power SYBR Mix (Applied Biosystems, Foster City, USA), 2 µl of the diluted cDNA, 4 pmol of each of forward and reverse gene-specific primers and ultrapure water up to 10 µl. RT-qPCR reactions were performed as follow: one step thermal cycle of 2 min at 95 °C, and then 40 cycles of 30 s at 95 °C and 30 s at 60 °C. A melting curve analysis was carried out to assess primer specificity and product quality by step-wise denaturation of the PCR product with the following conditions: a final step of 15 s at 95 °C, 1 min at 60 °C and then the fluorescence measured from 60 to 95 °C at each 0.7 °C increment of temperature.

The experiment was performed using biological triplicate samples for each condition tested. Two technical replicates were used for each biological replicate and the average Cq values (Cycle quantification) were used for quantification.

### Data analysis

A pool of cDNA from 36 samples was used to carry out the RT-qPCR reactions to estimate the efficiency of each primer pair using the LinRegPCR software version 2015.0^39^. This program uses non-baseline corrected data, performs a baseline correction on each sample separately, determines a window-of-linearity and then uses linear regression analysis to fit a straight line through the PCR data set from which the PCR efficiency of each individual sample is calculated. The primer efficiency is calculated based on slope of the line (E = 10^−1/slope^), considering an ideal value range (1.8 ≤ E ≤ 2) and correlation (R ≥ 0.995)^40^.

The expression stabilities of the nine candidate genes was analyzed by RefFinder^8^ a web-tool that use four different algorithms geNorm^5^, NormFinder^4^, BestKeeper^6^ and the comparative ΔCt method^7^ all together to establish a compreensive rank of gene stability. A score is then assigned to each gene considering the rankings of the various algorithms employed.

### Reference Gene Validation

In order to determine whether the choice of the reference genes ranked by RefFinder affects the normalization of target genes, the same cDNA samples used for the stability analysis of the reference genes were also analysed for the expression of the ribulose-1,5-bisphosphate carboxylase/oxygenase (*rbCl*) under drought, cold and heat stresses. The ranking of all the references genes, from the most stable to the less stable according to the RefFinder analysis, was used to normalize the *rbCl* expressions. The relative expression profile of *rbCl* was obtained according to the 2^−ΔΔCt^ method^41^. Plants grown without stresses treatments (controls) were selected as calibrator to calculate the ΔΔCt.
